# Macrophage-Centric Biomaterials for Bone Regeneration in Diabetes Mellitus: Contemporary Advancements, Challenges, and Future Trajectories

**DOI:** 10.7759/cureus.66621

**Published:** 2024-08-11

**Authors:** Yiyan Yang, Xiaoli He, Zhihe Zhao, Jianru Yi

**Affiliations:** 1 Department of Orthodontics, State Key Laboratory of Oral Diseases and National Clinical Research Center for Oral Diseases, West China School of Stomatology, Sichuan University, Chengdu, CHN

**Keywords:** osteogenesis, biomaterials, bone regeneration, macrophage, diabetes mellitus

## Abstract

Increased susceptibility to bone fragility and the diminution of bone regenerative capacity are recognized as significant and frequent sequelae of diabetes mellitus. Research has elucidated the pivotal role of macrophages in the pathogenesis and repair of diabetic bone defects. Notwithstanding this, the therapeutic efficacy of traditional interventions remains predominantly inadequate. Concomitant with substantial advancements in tissue engineering in recent epochs, there has been an escalation in the development of biomaterials designed to modulate macrophage activity, thereby augmenting osseous tissue regeneration in the context of hyperglycemia. This review amalgamates insights from extant research and delineates recent progressions in the domain of biomaterials that target macrophages for the regeneration of diabetic bone, whilst also addressing the clinical challenges and envisaging future directions within this field.

## Introduction and background

Diabetes mellitus is a metabolic disease characterized by hyperglycemia and chronic inflammation. As of 2021, the prevalence of diabetes mellitus has reached 536.6 million worldwide, with an estimated increase to 783.2 million in 2045 [1]. The persistent hyperglycemic state and chronic inflammatory response contribute to a spectrum of complications, including cardiovascular diseases, nerve damage, chronic kidney diseases, and bone disorders, imposing a substantial burden on healthcare systems and patients [2,3].

Among the complications, diabetic bone diseases have garnered considerable attention. These maladies conditions manifest as reduced bone density, long-term nonunion of bone defects, and alterations in bone microstructure [4,5]. The underlying mechanisms are multifaceted, involving impaired osteogenic capacity, heightened osteoclastic activity, and unremitting inflammatory processes [6]. Prolonged hyperglycemia inhibits the migration and proliferation of bone marrow mesenchymal stem cells (BMSCs), diverting them toward adipogenic lineage rather than osteogenic lineage, and promoting BMSC senescence and apoptosis [7]. Moreover, the dysregulation of osteoblasts and osteoclasts due to hyperglycemia disrupts bone homeostasis [8]. The high glucose environment further destabilizes immune equilibrium, culminating in chronic inflammation that impedes bone repair processes [5]. Additionally, the hyperglycemic microenvironment at injury sites exacerbates susceptibility to bacterial infections, further complicating the therapeutic landscape of diabetic osteopathy [9].

Macrophages, a subset of leukocytes, actively participate in both innate and adaptive immunity. These versatile immune cells are instrumental in orchestrating inflammation and bone remodeling during reparative phenomena. Nonetheless, diabetes mellitus precipitates aberrant macrophage activation, engendering exacerbated inflammatory reactions that hinder osteogenic repair mechanisms [10]. Indeed, several studies have underscored that strategic modulation of macrophage functionality can significantly ameliorate bone defect rectification under hyperglycemic conditions [11-13]. Hence, macrophage immunomodulation has been identified as a salient therapeutic avenue for the enhancement of diabetic bone repair [14].

The preceding decades have witnessed substantial evolution and integration of biomaterials in the realm of bone regeneration, attributable to their advantageous physicochemical attributes [15]. These materials eclipse conventional therapeutic modalities by precise targeting capabilities, minimized dosing requirements, and attenuated adverse effects [15]. Recognizing the pivotal role of macrophages in diabetic bone diseases, burgeoning research endeavors have concentrated on the synthesis of biomaterials that engage macrophages to expedite bone restoration in the diabetic context [16]. This review endeavors to encapsulate the contemporary advancements in macrophage-centric biomaterials for diabetic bone regeneration, whilst concurrently contemplating the challenges inherent in their application and projecting prospective trajectories in this burgeoning field.

## Review

This review employed a comprehensive search approach to identify all relevant articles published in multiple databases including PubMed, Scopus, Science Direct, and Web of Science, using the keywords “biomaterials”, “macrophages”, “bone regeneration”, and “diabetes”. To enhance the search, the most frequently used phrases in publications on this topic were combined with Medical Subject Headings (MeSH) terms. Inclusion criteria were: (i) biomaterials that relate to macrophages, and (ii) designed for bone regeneration in diabetes mellitus. All irrelevant articles were excluded. Manual searches were also performed by cross-checking the references of the included literature to identify potentially overlooked publications during the electronic search.

The critical role of macrophages in diabetic bone regeneration

Macrophages in Normal Bone Regeneration

Macrophages are dichotomously categorized into two principal phenotypes: the pro-inflammatory M1 and the anti-inflammatory M2 [17]. M1 macrophages are characterized by their secretion of inflammatory cytokines including tumor necrosis factor-alpha (TNF-α), Interleukin (IL)-1β, IL-6, and inducible nitric oxide synthase (iNOS), which, upon sustained exposure, can precipitate immunopathological responses and consequent tissue detriment [18]. Conversely, M2 macrophages are distinguished by their release of anti-inflammatory agents like IL-10 and TGF-β1, which are vital for stem cell viability during tissue repair [19].

In the context of bone homeostasis, macrophages are pivotal, executing phagocytosis and cytokine production [20]. They modulate inflammatory reactions, osteogenesis, and osteoclastogenesis throughout the bone regeneration process [21], with behaviors varying dynamically at different stages of bone repair due to the highly dynamic extracellular microenvironment [22]. During the initial inflammation phase, immune cells rapidly migrate to sites of bone injury. Here, inflammatory mediators stimulate monocytes and resident macrophages to differentiate into the M1 phenotype [22]. Concurrently, the hematoma surrounding the injury fosters a hypoxic, acidic environment conducive to M2 polarization and the osteogenic and chondrogenic differentiation of bone marrow mesenchymal stromal cells (BMSCs) [23,24]. Typically, a phenotypic shift from M1 to M2 occurs within three to four days after injury, whereas persistent M1 activation can lead to prolonged inflammation at the injury site, ultimately impeding bone healing [25]. In the subsequent primary callus stage, BMSCs differentiate into fibroblasts, chondroblasts, and osteoblasts, facilitated by M2-derived cytokines like IL-4 and IL-10. Simultaneously, M1-produced cytokines such as IL-6 and TNF-α instigate angiogenesis by promoting endothelial cell migration and proliferation [26,27]. As the macrophage phenotype transitions towards M2, anti-inflammatory cytokine expression increases, consolidating angiogenesis and orchestrating extracellular matrix (ECM) assembly through pericyte recruitment and differentiation [28].

In the final stage, the callus is progressively replaced by new bone tissue, necessitating a delicate equilibrium between bone formation and resorption, orchestrated by macrophages [29]. M1 macrophages impede BMSC osteogenic differentiation via MAPK pathways [30], and impede ECM secretion and mineralization through IL-1β and TNF-α [31]. Simultaneously, M1 macrophages promote osteoclastogenesis by activating JAK2 and nuclear factor kappa-light-chain-enhancer of activated B cells (NF-κB) pathways through IL-6 and TNF-α, respectively [32,33]. Conversely, M2 macrophages inhibit early-stage osteoclastogenesis by interfering with NF-κB binding, and contribute to osteogenesis [34,35]. In return, skeletal stem cells mitigate M1 macrophage functions and promote the production of anti-inflammatory cytokines such as IL-10 from M2 macrophages by releasing prostaglandin E2 [36,37].

Collectively, M1 macrophages are instrumental in the initial phase of bone repair, promoting regeneration, while the M2 phenotype gains prominence in subsequent stages. The intricate and harmonious interplay among macrophages, osteoblasts, and osteoclasts ensures the successful restoration of normal bone tissue structure and function.

Macrophage in Diabetic Bone Regeneration and Targeting Strategies

Macrophages, owing to their remarkable phenotypic plasticity, exhibit adaptability to diverse stimuli within the bone microenvironment, resulting in distinct functional polarization. These phenotypic and functional shifts in macrophages are significant in diabetic bone metabolism [38]. Hyperglycemic conditions induce a metabolic pivot towards glycolysis in monocytes and macrophages, leading to an increase in reactive oxygen species (ROS) production [39]. ROS serves as a critical mediator for pro-inflammatory pathway activation and can instigate M1 polarization through epigenetic reprogramming [39]. An imbalanced M1/M2 macrophage ratio disrupts osteogenesis and promotes osteoclastogenesis, thereby impeding bone regeneration in hyperglycemic states. Additionally, heightened glucose levels enhance the expression of the glucose transporter type 1 sensor in macrophages, potentially inducing macrophage senescence. The accumulation of senescent cells and their associated secretory phenotype further exacerbates inflammatory bone loss in diabetes [40].

In light of their integral role in diabetic bone regeneration, macrophages have been identified as a promising therapeutic target to improve repair efficacy. Presently, two primary macrophage-centric strategies are being explored. The first involves modulating macrophage phenotypes at defect sites, with a focus on fostering M2 polarization and transitioning M1 macrophages towards an M2 phenotype [16], as such transitioning macrophages exhibit superior regenerative capabilities compared to those directly polarized to M2 from inactive monocytes [41]. However, M1 macrophages are essential for angiogenesis, and excessive M2 activation may result in fibrosis and pathological osteogenesis due to fibroblast cytokine secretion [36,42]. Thus, a strategic approach is required for the sequential activation of M1 and M2 phenotypes and the regulation of their ratio, as excessive inhibition of M1 macrophages in the early stage or insufficient activation of M2 macrophages in the later phase can compromise bone regeneration [43]. The second strategy aims to bolster the macrophage population at defect sites to counteract hyperglycemia-induced macrophage senescence and apoptosis. It has been proved that bone regeneration can be enhanced by augmenting the macrophage population through local injection of macrophage colony-stimulating factor (M-CSF) at fracture sites [44,45]. Furthermore, direct delivery of monocytes or pre-polarized macrophages to injury areas has also shown favorable effects [21].

Macrophage-related biomaterials for diabetes mellitus

Although conventional therapeutic methods demonstrate efficacy under physiological conditions, they fall short of achieving satisfactory outcomes for diabetic bone defects. This limitation arises due to the complicated microenvironment in diabetes mellitus and altered macrophage niches [14]. Biomaterial-based therapies offer a promising avenue for enhancing bone reconstruction in diabetes. They have the potential to address two major challenges faced by traditional strategies: the lack of control over the maintenance of macrophage phenotype, and the limited retention of macrophages at injury sites [46].

Metal and Metal Alloy Biomaterials

Metals and metal alloys find extensive use in load-bearing bone applications owing to their remarkable strength and stiffness. Titanium (Ti) and its alloys are particularly prevalent in bone repair implants [47,48]. Surface modifications at the nanoscale and functional coatings enhance the regulatory effects of Ti-based materials on macrophages. For instance, coating Ti-based scaffolds with piezoelectric Barium Titanate promoted anti-inflammatory polarization of macrophages and improved bone repair efficacy [49]. Similarly, aspirin/poly(lactic-co-glycolic acid) (PLGA)-coated Ti-based substrates exhibited enhanced M2 protein expression, reduced M1 protein expression, and greater osteogenic potential both in vitro and in vivo [50].

Natural Polymers

Macrophage-targeting natural polymers primarily originate from the ECM and other biological sources. These polymers offer inherent biocompatibility and anti-inflammatory effects, leading to the development of preclinically applicable biomaterials with optimized physicochemical properties [51]. Decellularized ECM scaffolds, which maintain native ECM components and structure [16], significantly encourage M2 polarization, fostering an immune-supportive environment [52]. This effect may be attributed to the rheological properties and protein components of the scaffold [52]. It’s also noteworthy that thorough decellularization is crucial, as cellular remnants and mitochondria can trigger pro-inflammatory responses [53]. Due to the immunogenic blocks of β-D-mannuronic acid, alginate is capable of regulating macrophage phenotypes, cytokine release, and phagocytosis through increasing Toll-like receptor 4 expression and activating the NF-κB and p38 mitogen-activated protein kinase (MAPK) signaling pathways [54,55]. Sulfated alginate, in particular, promotes M2-like polarization of human THP-1 monocytes and reduces pro-inflammatory cytokine production [56]. Polyguluronate liposomes fabricated using α-L-guluronic acid derived from alginate have been also confirmed to enhance macrophage-stimulating activity [57].

Hydrogel scaffolds made from natural polymers have also been explored. A novel scaffold enriched with platelet lysate demonstrated sustained bioactive component release, promoting M2 macrophage migration and polarization, and thereby increasing anti-inflammatory cytokine production [58]. Furthermore, scaffolds incorporating vesicles from red blood cell membranes have been shown to attract M2 macrophages, maintaining their presence for one month post injection [59].

Synthetic Polymers

Synthetic polymers represent a class of intentionally designed biomaterials with diverse structures and customized properties. Notable examples include polylactic acid (PLA), polyglycolic acid (PGA), PLGA, and polycaprolactone (PCL) [60]. In the context of bone regeneration, synthetic polymers are frequently employed to construct scaffolds, serving as structural support for defect areas and platforms for localized drug delivery [61].

Recent advancements have seen the development of polymers that can influence macrophage activity. For instance, a particular copolymer, poly(octanediol-citrate-polyglycol) (POCG), has demonstrated the ability to suppress proinflammatory cytokine expression and polarize macrophages toward the M2 phenotype [62]. Additionally, polymeric electrospun fibers have also exhibited immunomodulatory effects on macrophages, leading to high-quality bone regeneration in both in vitro and in vivo experiments [63].

Composite Biomaterials

Given the intricate and hierarchical structure of human bones, a single biomaterial may fall short of meeting the requirements for effective bone structure and function restoration. Consequently, efforts have been directed toward developing composite biomaterials that integrate multiple components. A common strategy involves combining polymers with inorganic materials to create scaffolds [64]. Inorganic particles such as silica nanoparticles and bioceramic nanoparticles can serve as drug delivery carriers to modulate macrophage behavior [65,66]. Another method involves the use of metal-organic frameworks (MOFs), which exhibit high specific surface areas and tuneable pore structures, enabling efficient capture, transport, and release of regulatory agents [67]. These properties make MOFs promising candidates for regulating macrophage functions and promoting bone repair.

Recent research progress

With the development of biomaterials, intelligent responsive biomaterials that are capable of sensing microenvironment changes, particularly glucose levels, have emerged as a focal point in diabetic bone regeneration [68]. Li et al. engineered a hydrogel with a dual-logic response mechanism, composed of reversibly cross-linked poly(vinyl alcohol) and a colloidal network formed from gelatin nanoparticles [69]. This hydrogel demonstrated the capacity for intelligent modulation of drug release contingent upon glucose, ROS, and matrix metalloproteinase levels, thereby facilitating macrophage polarization and enhancing bone repair under diabetic conditions. Wang et al. developed a glucose-sensitive system capable of delivering anti-TNF-α antibodies through a quaternized chitosan and collagen scaffold [70]. This system mitigates M1-related local inflammation under long-term hyperglycemic conditions. Furthermore, Zheng et al. constructed a pH-responsive nanoplatform comprising an IL-4-loaded gallic acid-magnesium-based MOF core and a biodegradable calcium phosphate shell [71]. When integrated into collagen scaffolds, this nanoplatform effectively safeguarded bioactive factors, exhibiting controlled degradation in response to acidic conditions, thereby fostering M2 macrophage polarization.

Furthermore, Kang et al. introduced an innovative magnetic-controlled system for remote manipulation of macrophage behavior [72]. By conjugating Arg-Gly-Asp (RGD)-grafted gold nanoparticles (GNPs) to a glass substrate and pairing them with magnetic nanocages via a flexible linker, they created a heterodimeric nanostructure. This configuration allowed for the precise control of RGD exposure, enhancing macrophage adhesion and M2 polarization through rho-associated protein kinase signaling pathways, while simultaneously suppressing M1 polarization, both in vitro and in vivo.

Three-dimensional (3D) bioprinting holds immense promise for constructing precisely controlled structures with tailored mechanical and biological properties [61]. Unlike other technologies, it enables the simultaneous fabrication of structures composed of biomaterials and cells [61]. The key component in 3D bioprinting is the bioink, which encompasses cell-loaded hydrogels, decellularized ECM-based solutions, and cell suspensions [73]. Sun et al. demonstrated the efficacy of a 3D bioprinted scaffold using a modified bioink containing gelatin, gelatin methacryloyl, and four-arm poly(ethylene) glycol (PEG) acrylate [74]. Loaded with BMSCs, RAW264.7 macrophages, and mesoporous silica nanoparticles carrying BMP-4, this scaffold significantly promoted M2 polarization and osteogenic differentiation of BMSCs upon implantation in a diabetic mellitus context. The latest biomaterials targeting macrophages for bone regeneration in diabetes are described in Table 1.

**Table 1:** The latest progress in biomaterials targeting macrophages for bone regeneration in diabetes TNF-α: tumor necrosis factor-alpha; IL: interleukin; Mg: magnesium; Ca: calcium; ROS: reactive oxygen species; MMP: matrix metalloproteinases; BMP2: Bone morphogenetic protein-2; RGD: arginine–glycine–aspartic acid; GelMA: gelatin methacrylate; PEG: poly(ethylene glycol); MSN: mesoporous silica nanoparticle; BMSC: bone marrow mesenchymal stromal cell

Biomaterials	Functional characteristics	Effects on macrophages	Advantages	Disadvantages
Glucose-sensitive scaffolds	Accelerated delivery rate of TNF-α antibody with the increase of glucose concentration.	Inhibiting M1 polarization.	1. Improved efficiency of drug release. 2. Honeycombed structure with pore shape and porosity suitable for bone regeneration.	The drug content of the system still needed to be improved.
pH-responsive nanoplatforms	Slowly degrading and releasing gallic acid, IL-4, Mg^2+^, and Ca^2+^ ions with the decrease of pH.	Enhancing M2 polarization.	1. Protecting bioactive factors loaded inside. 2. Moderate and sustained delivery rate.	The surface of nanoplatform hybrid scaffold was uneven.
Dual-logic-based system	Responding to the level of glucose, ROS, and MMPs, and determining the release time and types of specific cargos (IL-10 and BMP-2).	Regulating macrophage polarization by remodeling the mitochondria-related antioxidative system.	1. Possessing both diagnostic and basic therapeutic logic. 2. Accurately regulating IL-10 and BMP-2 delivery to match the period of macrophage polarization.	Not effective enough in severe diabetes with high glucose fluctuation.
Magnetic-controlled system	Magnetically manipulating nanoscale displacement of magnetic nanocage to reversibly uncage or cage RGD.	Promoting the adhesion and M2 polarization, and inhibiting M1 polarization.	1. Working as a single magnetic domain to promote osteogenesis without an external magnetic field stimulus. 2. Achieving the remote and reversible manipulation on the uncaging of RGD.	The system was not stable enough and likely to be interfered with by the external magnetic fields.
3D bio-printed scaffolds	1. Constructed by highly biocompatible GelMA/gelatin/PEG/MSN composite bioinks and loaded with BMSCs, macrophages, and mesoporous silica nanoparticles carrying BMP-4. 2. The sustained release of BMP-4 with the biodegradation of materials.	Promoting M2 polarization.	1. Precisely controlled shapes and structures. 2. Personalized mechanical and biological properties. 3. Containing biomaterials and cells simultaneously	Manufacturing processes placed high requirements on instruments.

Design strategies and functional mechanisms of macrophage-related biomaterials

The interaction of macrophages with these implanted materials is a complex and dynamic process. It initiates with the host’s primary immune response, marked by the sequential activation of M1 and M2 macrophage phenotypes [75]. The biomaterials subsequently assume a pivotal role in directing the functional transitions or stabilization within the macrophage contingent to establish the anti-inflammatory and pro-regenerative microenvironment. The bioactive molecules and pharmacological agents liberated from the biomaterials can directly govern the phenotypic and functional directives of macrophages, and the physicochemical properties of the biomaterials influence macrophage behaviors, including adherence, phagocytosis, plasticity, and apoptosis. In due course, these materials are subject to either complete or partial biodegradation via macrophage phagocytosis and/or degradation mediators released from foreign body giant cells (FBGCs) [76].

Biomaterials as Macrophage-Regulatory Delivery Systems

The use of biomaterial-based local delivery systems remains to be the forefront of bone regeneration strategies. These systems are designed to control the release of regulatory molecules, encompassing cytokines, pharmaceuticals, nucleic acids, and proteins. Some strategies are specifically tailored to administer pre-polarized macrophages or BMSCs directly [14,16].

One notable example is the localized administration of IL-4 to augment bone repair by upregulating the M2 macrophage phenotype in a dose-dependent manner [77]. Notably, biomaterial-based strategies guarantee a mild yet sustained release of IL-4, as well as avoiding the prolonged M1 phenotype phase caused by over-elevated doses of IL-4 [78]. The nanofibrous heparin-modified gelatin microsphere can improve IL-4 stabilization and prolong IL-4 release to modulate macrophage polarization from M1 to M2 phenotype, promoting bone regeneration in diabetes mellitus [79]. Similarly, the gelatin/β-tricalcium phosphate scaffold with sustained release of IL-4 to induce M2 polarization enhanced tooth extraction socket healing in diabetes [80]. Moreover, the local and sustained release of IL-10 has also been achieved in several biomaterial-based strategies, such as the physically crosslinked DNA hydrogel and the injectable self-assembling peptide hydrogel, which effectively promote the transition from M1 to M2 phenotype and diabetic bone reconstruction [81,82]. Beyond IL-4 and IL-10, other members of the TGF superfamily, such as TGF-β3, BMP-2, and BMP-4, are involved in regulating macrophages for bone regeneration in diabetes as well, making them potential targets in future research on biomaterial-based therapy [83,84].

Owing to their distinctive impact on macrophage behavior and function, metal ions have been critical elements in the design of biomaterials for bone repair in diabetes [85]. For instance, the prolonged and gradual release of Mg^2+^ ions from HA/MgO nanocrystal-based hybrid hydrogels markedly enhances bone regeneration related to diabetes by downregulating M1 macrophage infiltration and hastening the secretion of osteoblast-inducing factors like BMP-2 and IL-1RA from macrophages [86]. In addition, the local administration of specific pharmaceuticals constitutes an efficacious strategy. Sitagliptin, commonly prescribed for type 2 diabetes, promotes M2 polarization, curtails M1 phenotype stimulation, and diminishes IL-1β, TNF-α, and IL-6 secretion induced by lipopolysaccharide (LPS), thereby enhancing bone healing in diabetic patients [87-89]. Sitagliptin also reduces the mRNA expression of iNOS, IL-12 p35, and IL-12 p40 in macrophages, forestalling the escalation of inflammatory responses and subsequent tissue damage [89].

More recently, there has been the development of biomaterials that are specifically designed to sequentially release disparate regulatory molecules to govern macrophage recruitment and M1/M2 states at various stages [43]. The most prevalent approach involves the initial release of interferon-gamma (IFN-γ) to induce the M1 phenotype, followed by the subsequent delivery of IL-4/13 to effectuate M2 polarization [43,90]. To ensure adequate macrophage infiltration, MCP-1 is incorporated for release during the early inflammatory phase [91]. However, most extant sequential delivery systems have yet to achieve a clear demarcation between the two release phases, potentially inducing mixed phenotypes and disrupting the functionality of various cells [43]. There is an ongoing need to develop more sophisticated methodologies to precisely control the timing and dosage of release.

**Figure 1:**
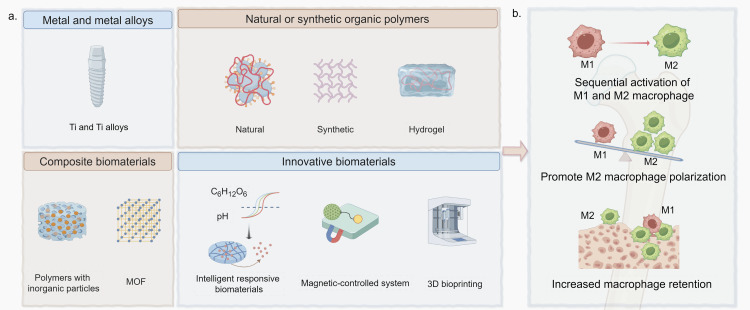
Macrophage-related biomaterials for diabetic bone regeneration (a) Common types of macrophage-related biomaterials; (b) Functional mechanism of macrophage-related biomaterials. Macrophage-related biomaterials are designed to achieve sequential activation and maintenance of M1 and M2 phenotypes, enhance M2/M1 ratio by promoting M2 macrophage polarization, and increase macrophage retention at defect sites, thereby enhancing bone regeneration in hyperglycemia. Image Source: Authors

Due to the complex and changeable microenvironment, the hindered recruitment of monocytes to defect sites, and the dysfunctional polarization due to epigenetic alterations in diabetes, strategies that solely depend on the patient’s endogenous macrophages may fail to achieve optimal therapeutic results [92]. Hence, biomaterials that are devoted to directly delivering pre-polarized M2 macrophages or BMSCs to defect sites emerge as a promising approach, particularly for the elderly demographic [46]. Nonetheless, this strategy necessitates further exploration regarding its safety and feasibility. Figure 1 shows the common types of macrophage-related biomaterials and their functional mechanism.

Biophysical Properties of Biomaterials With Regard to Macrophages

Macrophages exhibit sensitivity to the biomechanical alterations in substrate stiffness, which can manifest in variations in their morphological structure, migratory patterns, polarization, and functional capacities [16,93]. Presently, no consensus regarding the optimal stiffness parameters of various biomaterials for governing phenotypes has been reached. Some studies reported that an escalation in stiffness favors M1 polarization [93], while others have demonstrated contrary outcomes [94,95].

The topographical attributes of biomaterials are also critical determinants. Surfaces with greater roughness have been shown to exert a pronounced anti-inflammatory influence on macrophages [96], and microscale crumpled topographies have been significantly associated with an increase in M2 polarization, as opposed to nano- and submicroscale structures [97]. Intriguingly, surfaces with lower roughness at the nanoscale also promote M2 polarization and the release of anti-inflammatory cytokines [98]. The material surface patterns are also crucial. Micro-stripe patterned substrates are inclined to foster an M2 phenotype, with grooves guiding macrophages to align along the pattern direction, thereby enhancing IL-10 secretion and diminishing TNF-α expression [99,100].

Additional factors influencing macrophage behavior include the dimensions and geometry of pores and particles, porosity, particle concentration, and molecular weight of biomaterials. The enlarged pore sizes and higher porosity tend to induce a rounded macrophage morphology and favor M2 polarization [101,102]. Nano-sized particles, in comparison to micro-sized ones, significantly elevate the population of M2 macrophages and reduce the expression of inflammatory genes [103,104]. Within the nanometer scale, larger particle sizes may further enhance M2 polarization [105,106]. Spherical particles demonstrate a superior rate of phagocytosis and are more conducive to M2 polarization, relative to other geometries such as needles, rods, and worm-like forms [107-109]. Moreover, an increase in particle concentration has been found to induce an M1 phenotype, leading to oxidative stress and apoptosis [110]. Lower molecular weights tend to incite an M1 phenotype, whereas higher molecular weights are associated with M2 polarization [111].

Biochemical Properties of Biomaterials With Regard to Macrophages

Distinct functional groups within biomaterials have been empirically established to elicit specific responses in macrophages. Instructive consideration includes the involvement of the oxygen functional groups for greater macrophage adhesion and activation [112], and the introduction of amine to exert a pronounced anti-inflammatory effect on LPS-activated macrophages through the upregulation of autophagy pathway constituents [113,114]. Moreover, the catechol moieties group on biomaterial exhibits exceptional ROS scavenging capabilities, which significantly bolster the secretion of osteogenesis-related cytokines by M2 macrophages and inhibit M1 macrophage activity, thus expediting the healing of diabetic periodontal bone lesions [115].

In the context of osseous regeneration, the piezoelectric response and bioelectric transduction at the site of repair are imperative [116,117]. The localized reinstatement and conveyance of electrical signals are known to modulate the M2/M1 macrophage ratio under diabetic conditions [118]. Biomaterial surfaces with a positive charge are conducive to M2 polarization, while those with negative or neutral charges are inclined to induce an M1 phenotype [118,119]. Piezoelectric materials are capable of mimicking the electrical microenvironment, thereby inhibiting M1 polarization prompted by elevated glucose levels and facilitating the transition of macrophages from an M1 to an M2 phenotype [49].

The hydrophilic and hydrophobic characteristics of materials also exert a profound influence on macrophage adhesion, phenotype, and phagocytic activity. Surfaces modified to increase hydrophobicity promote macrophage adhesion and proliferation and suppress the pro-inflammatory response of M1 macrophages [120]. Interestingly for titanium materials, hydrophilic surfaces are associated with M2 polarization under both physiological and diabetic conditions [96,121,122].

Challenges and future perspectives

Adverse Immune Reactions Caused by Implanted Biomaterials

Macrophages play a dual role, not only participating in bone repair processes but also contributing significantly to foreign body reactions, a major challenge in the clinical application of biomaterials [123,124]. When phagocytosis of materials is frustrated, macrophages fuse to form FBGCs [124], which will release degradation mediators such as ROS and degradative enzymes, leading to an acidic environment at the biomaterial-tissue interface [124]. The degradation process may cause embrittlement, fragmentation, and damage to the biomaterial. Furthermore, degradation byproducts like lactic acid and carbon dioxide can exacerbate chronic inflammation [125]. In the late stages of the foreign body response, implanted biomaterials are encapsulated by a dense layer of avascular fibrotic tissue, which primarily forms due to myofibroblasts stimulated by TGF-β released from FBGCs [126]. The fibrous capsule isolates biomaterials from surrounding tissues, impeding their regulatory functions and causing discomfort and pain for patients [124]. These understandings of the intricate role of macrophages in foreign body reactions offer novel avenues to modulate biomaterial properties and mitigate adverse effects [127], especially in the context of diabetes mellitus.

Determining the Actual Effects of Biomaterials on Macrophages in Vivo

Though numerous studies have investigated the immunomodulatory effects of distinct biomaterials, drawing generalized conclusions remains challenging due to disparities in experimental conditions, including variations in cell sources, biomaterial concentrations, and culture duration [16]. Particularly, most of these experiments were conducted in vitro and over relatively short time frames, which may not fully align with the dynamic and variable conditions encountered by cells in vivo [128]. Furthermore, macrophages in vivo exhibit a spectrum of diverse phenotypes, which do not always correspond precisely to those induced by specific stimuli in vitro [128,129]. Meanwhile, human macrophage behavior is influenced by individual medical history, microbiome composition, and circadian rhythms, making accurate prediction of their responses challenging [130,131].

Another difficulty in the clinical application of macrophage-targeting biomaterials arises from disparities between mouse and human immune cells [132]. For instance, typical M1 and M2 markers in rodent models, such as iNOS and arginase 1, are not expressed in human macrophages [133], and the regulatory pathways controlling IL-10 and TGF-β1 expression differ between human and murine macrophages [133]. Another in vitro study observed that IL-4 and IL-13 upregulated the expression of IL-10 only in murine macrophages, while LPS and IFN-γ promoted vascular endothelial growth factor A (VEGFA) secretion merely from human macrophages [134,135]. Besides, macrophages from different origins may exhibit varying responses to identical physical cues. When cultured on a stiff substrate, human macrophages exhibited a greater increase in elasticity compared to RAW264.7 macrophages [136]. This discrepancy likely results from the stronger mechanical loading experienced by human macrophages in vivo, rendering them more sensitive to physical cues than RAW264.7 macrophages [137]. Despite considerable advancements in developing humanized mice with functional human immune systems, the high cost restricts their widespread application in experimental studies [138]. Hence, this issue remains to be further investigated.

The interaction between biomaterials, macrophages, and the skeletal system

In the past decades, extensive evidence has investigated the interplay between biomaterials, the skeletal system, and the immune system [22,139,140]. These systems dynamically modify their properties in a reciprocal manner, creating a feedback loop that eventually achieves equilibrium [141]. On this issue, further research is imperative to unravel the impact of the ever-changing microenvironment surrounding biomaterials on immune responses and osteogenesis processes. Moreover, understanding how immune cell behavior, particularly that of macrophages and stem cells, influences the properties and efficacy of biomaterials will yield precise guidelines and promising directions for designing and modifying biomaterials to treat diabetic bone defects.

Besides, while M2a macrophages are widely recognized as pivotal in tissue repair processes, the roles and temporal behaviors of other M2 subtypes remain to be clearly defined. Some studies have suggested that IL-10-stimulated M2c macrophages exhibit heightened activity during the early stages of tissue regeneration and may contribute to angiogenesis [142-144]. To advance our understanding, comprehensive investigations employing appropriate M1 and M2 markers, along with relevant gene analyses using diverse analytical methods as well as high-throughput analysis platforms, can provide critical insights into the interactions between different macrophage subtypes and biomaterials in both in vitro and in vivo settings, under physiological or pathological conditions [145].

Multidisciplinary integration

In recent years, significant strides have been made across diverse interdisciplinary domains pertaining to biomaterial research, significantly propelling the advancement and practical implementation of biomaterials for bone regeneration. More recently, machine learning and artificial intelligence have exhibited unique advantages in predicting interactions between macrophages and implants. For instance, Rostam et al. conducted a study involving a polymer library, leveraging machine learning techniques to establish connections between chemical descriptors of polymer structures and markers indicative of macrophage inflammatory responses [146]. Their findings highlighted a robust correlation between macrophage behavior and specific chemical features such as alkoxy groups and methacrylamide molecular fragments. Fostering greater multidisciplinary integration proves favorable in elucidating the underlying mechanisms governing macrophage behavior within biomaterial-based strategies for diabetic bone repair.

## Conclusions

Characterized by high glucose and chronic inflammation, diabetes mellitus is one of the major metabolic diseases threatening global human health. Due to the complex and dynamic microenvironment in hyperglycemia conditions, treatment strategies for diabetes bone diseases require to be highly precise and effective, in regard to which biomaterials possess unique advantages by providing structural support for defect sites and serving as delivery platforms for drugs, bioactive molecules, and cells. Besides, as macrophages play a pivotal role in both the pathogenesis and repair of diabetic bone defects by influencing osteoimmune environments, numerous biomaterials have been designed with a macrophage-targeting approach.

These designs involve the controlled release of regulatory components and modulation of physicochemical properties, aiming to achieve sequential activation and maintenance of M1 and M2 phenotypes, enhanced M2/M1 ratio, and increased macrophage retention at defect sites, thereby enhancing bone regeneration in hyperglycemia. Despite substantial progress, overcoming adverse immune reactions caused by implanted biomaterials remains a challenge, necessitating a deeper understanding of interactions between biomaterials, the immune system, and the skeletal system in vivo. Furthermore, research on fabrication and modification technologies is necessary to regulate macrophage behaviors and functions more accurately and efficiently for bone regeneration in diabetes.
